# Loss of 5-Hydroxymethylcytosine as an Epigenetic Signature That Correlates With Poor Outcomes in Patients With Medulloblastoma

**DOI:** 10.3389/fonc.2021.603686

**Published:** 2021-02-24

**Authors:** Fu Zhao, Zhi-Wei Zhang, Jing Zhang, Shun Zhang, Heng Zhang, Chi Zhao, Yang Chen, Lin Luo, Wei-Min Tong, Chunde Li, Yamei Niu, Pinan Liu

**Affiliations:** ^1^ Department of Neurosurgery, Beijing Tian Tan Hospital, Capital Medical University, Beijing, China; ^2^ Department of Neural Reconstruction, Beijing Neurosurgical Institute, Capital Medical University, Beijing, China; ^3^ Department of Pathology, Institute of Basic Medical Sciences, Chinese Academy of Medical Sciences and Peking Union Medical College, Beijing, China; ^4^ Neuroscience Center, Chinese Academy of Medical Sciences, Beijing, China; ^5^ Department of Neuro-Oncology, Sanbo Brain Hospital, Capital Medical University, Beijing, China; ^6^ Institute of Medicinal Biotechnology, Chinese Academy of Medical Sciences and Peking Union Medical College, Beijing, China; ^7^ Department of Pathology, Beijing Neurosurgical Institute, Capital Medical University, Beijing, China

**Keywords:** medulloblastoma, 5-hydroxymethylcytosine, prognosis, Ki-67, epigenetics, immunohistochemistry

## Abstract

Medulloblastoma, as the most common malignant brain tumor in children, exhibits highly dysregulated DNA methylation. The novel epigenetic marker—5-hydroxymethylcytosine (5hmC) plays essential role in gene regulation during brain development and in brain tumors. However, the biological and clinical implications of 5hmC in medulloblastoma are still unclear. Here, we detected global 5hmC levels in two independent medulloblastoma patient cohorts (discovery cohort: n = 81; validation cohort: n = 171) using ultra-high performance liquid chromatography-tandem mass spectrometry analysis. Immunohistochemistry was used to identify the cell proliferation and expression of Ten-eleven translocation 1 and 2 (TET1/2). The prognostic impacts of covariates on progression-free survival (PFS) and overall survival (OS) were evaluated using multivariate Cox hazards regression models. We observed that global 5hmC levels were decreased in medulloblastomas compared to normal cerebellums (*P* < 0.001). Multivariate analysis showed that low global 5hmC levels correlated with poor PFS and OS rates (discovery cohort: PFS: *P* = 0.003, OS: *P* = 0.002; validation cohort: PFS: *P* = 0.0002, OS: *P* = 0.001). Immunohistochemistry showed an inverse correlation between 5hmC score and Ki-67 index (*r* = -0.747, *P* < 0.0001). Moreover, 5hmC score in MB samples was associated with nuclear expression of TET1 (*r* = -0.419, *P* = 0.003) and TET2 (*r* = -0.399, *P* = 0.005) proteins. Our study demonstrates that loss of 5hmC is an epigenetic biomarker in medulloblastomas. Our results indicate that 5hmC could be a candidate prognostic indicator for improving survival prediction of risk stratification in patients with medulloblastoma.

## Introduction

Medulloblastoma (MB) is a malignant embryonal tumor of the cerebellum that represents over 20% of all pediatric central nervous system (CNS) neoplasm (1). Although conventional treatments have significantly improved outcomes in recent years, survivors are frequently left with devastating neurocognitive impairment and other sequelae following such therapy (2, 3). Therefore, the purpose for developing treatment strategies in MB is to increase the survival rates for high-risk patients, and to improve the quality of life of survivors by reducing the toxicity of treatment. However, the current criteria for risk stratification rely primarily on the patient age, the presence of metastases, the extent of resection (EOR), and the histopathological subtypes (4–7), which is insufficient for predicting the outcome. Recent advances have shown that MBs can be classified into at least four subgroups with distinct underlying biological and clinical features (8–11). Identifying molecular subgroups has strong potential for improving clinical management and provides a basis for investigating the biological consequences of subgroup-specific therapeutic applications (12–15). However, more reliable and practical prognostic biomarkers are still urgently needed to develop individualized treatment options for MB.

DNA methylation of the fifth position of cytosine (5mC, 5-methylcytosine) is acknowledged as an epigenetic mechanism and its alterations in genomic DNA are associated with tumorigenesis (16, 17). Recent studies demonstrate that 5-hydroxymethylcytosine (5hmC) is a necessary intermediate in the DNA passive demethylation process that catalyzed by the ten-eleven translocation (TET) protein family (18, 19). The TET family consists of three members—TET1, TET2, and TET3—which are responsible for the conversion of 5mC into 5hmC through Fe^2+^ and α-ketoglutarate-dependent dioxygenase activity (20). 5hmC is highly enriched in the human brain, and plays as a stable epigenetic modifier of gene expression during neuronal differentiation and development (21, 22), and as an important epigenetic mark in neurological disease (23). The depleted global 5hmC levels have been observed in brain tumors and strongly correlate with increased malignancy and poor prognosis (24–27). Moreover, the inhibition of TET activity or TET expression is also observed in many types of human cancer and relates to unfavorable outcome (19).

However, little is known regarding the biological function and tumorigenesis of 5hmC in MB. To investigate the clinical and biological implications of this new epigenetic biomarker in MB, we detected global 5hmC levels in two independent cohorts of patients with MB (n = 81; n = 171) using ultra-high performance liquid chromatography-tandem mass spectrometry (UHPLC-MS/MS) analysis. We assessed the prognostic value of global 5hmC levels on the progression-free survival (PFS) and overall survival (OS) for patients with MB.

## Materials and Methods

### Patient Cohorts

Patient with initial surgery for MB at Beijing Tian Tan hospital, Capital Medical University between 2003 to 2018 were included in the study. Tumor specimens were stored in liquid nitrogen or in formalin-fixed paraffin-embedded (FFPE) blocks at Beijing Neurosurgical Institute. Patients with complete clinical data (such as age, EOR, and survival), preoperative magnetic-resonance image (MRI) scans, and surgical tissues obtained during initial surgery (before radiation or any other adjuvant treatment) were included. Patients without complete clinical data, or without enough tumor samples for molecular classification and immunohistochemical (IHC) staining or lost to follow-up were excluded. The EOR was established based on surgeons’ reports and confirmed with postoperative enhanced T1-weighted MRI scans. This study was approved by the Ethics Review Board of Beijing Tian Tan Hospital (Capital Medical University; Approval Number: KY2018-020-01). All patients or families provided written informed consent. Normal cerebellums were provided by the Human Brain Bank (Chinese Academy of Medical Sciences & Peking Union Medical College), with the approval from the Institutional Review Board of the Institute of Basic Medical Sciences (Chinese Academy of Medical Sciences; Approval Number: 009-2014).

### Molecular Subgroup Analysis

Molecular classification (wingless [WNT], sonic hedgehog [SHH], Group 3, or Group 4) was established using three methods, as we described previously (28, 29): 1. Gene expression profiling of the samples was analyzed using Agilent Whole Human Genome Oligo Microarray Kit, 4×44K (Agilent Technologies, Santa Clara, CA, USA; GSE116028; n = 38). Data were extracted with Feature Extraction Software v10.7 (Agilent Technologies). Raw data were normalized by Quantile algorithm, Gene Spring Software v11.0 (Agilent Technologies). Genes with a fold change ≥ 2 and *P* < 0.05 were selected for further analysis. Pathway annotation was performed by Ingenuity Systems. 2. Twenty-two subgroup-specific signature genes were detected by the QuantiGene Plex Gene Expression Assay (QGP, version 2.0, Affymetrix, Santa Clara, CA, USA, n = 97). All of the experiments were performed following the user manual of QuantiGene Plex Assay (Panomics). Normalized expression ratios were generated by dividing the background-subtracted expression values by the geometric mean of the housekeeping genes. Unsupervised hierarchical cluster analysis was used to delineate distinct sample clusters. 3. The IHC staining was performed with four antibodies [β-catenin (1:100; ab610154, BD Transduction Laboratories), SFRP1 (1:2,000; ab4193, Abcam), NPR3 (1:200; ab37617, Abcam), and KCNA11:2,000; ab32433, Abcam), n = 117], as we described previously (28, 29).

### UHPLC-MS/MS Analysis

Absolute quantities of 5hmC were measured, as we previously described (30). Briefly, DNA isolation was performed using the Wizard^®^ Genomic DNA Purification Kit (A1620, Promega, Madison, WI, USA) according to the manufacturer’s protocol. DNA for each sample (1 μg) was denatured by heating at 100°C for 3 min and then digested by incubation at 42°C with nuclease P1 (2U, Sigma, N8630, Darmstadt, Germany) for 6 h. Subsequently, 1 U of alkaline phosphatase (Sigma, M183A) was added and incubated at 37°C for another 6 h. Finally, the sample was diluted to a total volume of 60 μl and filtered (0.45μm, PALL). Nucleosides were separated by UHPLC on a T3 column (Waters, 186003538) and detected using a triple-4 quadrupole tandem MS instrument (Waters, ACQUITY UPLC XEVO TQ-S). The mass/change (m/e) transitions of 228.4 to 112.2 (cytosine), 242.3 to 126.1 (mC), and 258.2 to 124.2 (hmC) were monitored and recorded. Quantification was performed in comparison with standard curves generated using pure nucleoside standards, which were run with the same batch of samples. 5hmC percentages were calculated using the formula: 5hmC% = M (5hmC)/(M [cytosine] + M [5mC] + M [5hmC]) × 100.

### Immunohistochemistry Analysis

Immunohistochemistry (IHC) staining for 5hmC, Ki-67, and TET1/2 was performed on FFPE sections, as previously described (29, 31, 32). Briefly, FFPE tissues were cut into 4-μm sections, followed by deparaffinization and rehydration using xylene and ethanol. Next, the slides were incubated in 3% hydrogen peroxide for 10 min in phosphate-buffered saline and then in blocking solution (CSA II Kit; Dako, Glostrup, Denmark) for 60 min at room temperature. The slides were incubated overnight with primary antibodies against 5hmC (1:800, ab214728, Abcam, US), Ki-67 (1:1,000, ab15580, Abcam), TET1 (1:1,000, HPA019032, Sigma, US), and TET2 (1:100, ab94580, Abcam). The number of pixels representing positively stained nuclei was detected using Image-Pro Plus image-analysis software (Media Cybernetics, Inc, MD, US). Positive staining was defined as a dark-brown staining pattern, confined to the nuclear region. For each sample, the mean value of ten snapshots was calculated to represent the percentage of positive cells. Ki67 index was calculated as the percentage of nuclear-positive cells in every 100 cells. For 5hmC staining, normal cerebellum and non-tumor cells (endothelial cells) in the microenvironment were used as a positive-control tissue. All IHC slides were separately reviewed by two senior neuropathologists. A final score for 5hmC staining was then calculated by multiplying the score of proportion of positively stained tumor cells (0%–100%) and the score of staining intensity (0, 1, 2, 3).

### Gene Expression Analysis

To evaluate the expression levels of 5hmC-related genes in MB, we downloaded normalized gene-expression data generated with the Removal of Unwanted Variation method from the Gene Expression Omnibus database (accession number GSE124814; https://www.ncbi.nlm.nih.gov/geo/), which included cerebellar data for 1350 patients with MB and 291 normal brain samples (33). Expression levels of the *TET1/2* genes and the isocitrate dehydrogenase 1 and 2 (*IDH1* and *IDH2*) genes were analyzed in MB and normal cerebellum samples.

We detected the RNA-expression levels of *TET1/2* in our MB samples using the QGP Assay. All samples were analyzed using a Luminex^®^ instrument, and gene-expression levels was normalized to the geometric mean of the expression for two housekeeping genes (*ACTB* and *GAPDH*).

### *TP53* Mutation Analysis

Mutations of *TP53* gene (exons 2 through 11) in SHH-MB was detected by Sanger sequencing, as previously described (34). Briefly, genomic DNA derived from FFPE samples was prepared with Wizard^®^ Genomic DNA Purification Kit (A1120, Promega, US) according to the manufacturer’s protocols. The PCR profile was performed as follows: 95°C for 2 min, 56°C for 1 min for 35 cycles. The final extension was added at 72°C for 10 min before storage at 4°C. The PCR products were loaded onto a 1% of agarose gel for electrophoresis. The reactions were analyzed by an automated Genetic Analyzer ABI 310 system (ABI, CA) according to the manufacturer’s instructions.

### Statistical Analysis

Significant differences between two groups were analyzed using Student’s t-test (two-tailed). Data are reported as mean ± standard deviation (SD). Relationships were evaluated by the Pearson correlation coefficient. For the survival analyses, OS was defined as the time from diagnosis until death, and PFS was defined as the time from the date of surgical resection until the date of tumor progression. Estimated 5- year OS and PFS were calculated using Kaplan–Meier analysis and data are reported as the mean ± standard error (SE). Patient cohorts were divided into two groups according to 5hmC levels. The optimal cut-off value was defined as the point with the most significant split in the discovery cohort (< 0.51 and ≥0.51) and the validation cohort (< 0.54 and ≥0.54) respectively using Cutoff Finder (http://molpath.charite.de/cutoff/index.jsp). Significant differences between survival curves were determined using the log-rank test. The discriminatory capacity of 5hmC-based classification was evaluated by Harrell’s C index and time-dependent receiver operating characteristic (ROC) curve analysis, using the “survival ROC” package in R software, as previously described (35).

Univariate and multivariable Cox proportional hazard regression to estimate hazard ratios (HRs) for PFS and OS, including 95% confidence intervals (CIs). The multivariate model was performed with backward stepwise selection. Variables with *P* value ≤ 0.10 in univariate analysis, including the global 5hmC levels (low *vs.* high), the molecular subgroup (WNT, SHH, Group 3, or Group 4), the pathological subtype (classic MB [CMB], desmoplastic/nodular MB [DNMB], or large cell/anaplastic MB [LC/AMB]), age (<3, 3–17, or ≥18 years old), metastatic status (yes *vs.* no), and receipt of craniospinal irradiation (CSI; yes *vs.* no) were subjected to the multivariate Cox analysis. Covariates (EOR and receipt of chemotherapy), which were deemed as important prognostic factors in previous studies (7, 15), were also included in the Cox regression models.

The nomograms were established based on the Cox model for predicting 3-, 5-, and 10-year PFS and OS rates of the validation cohort using the “rms” package (version 4-4.2) of R software. Calibration curves were generated to compare associations between the observed and predicted outcomes, as previously described (36). Time-dependent ROC curve analysis was used to evaluate the discriminative ability of the nomogram. All statistical analyses were performed using the SPSS Statistical Package software (version 23.0, IBM Inc., Chicago, US) or R software (version 3.4.3; http://www.r-project.org). *P* < 0.05 was considered to reflect a statistically significant difference.

## Results

### The Clinical Characteristics

This study included two non-overlapping patient cohorts (discovery cohort: n = 81; validation cohort: n = 171). The demographic, clinical, and molecular characteristics were shown in **Table 1**. The discovery cohort included 81 patients with a mean age of 11.1 ± 9.5 years (range: 1.5–49 years). The median follow-up period was 42.7 months (10–116 months), and the estimated 5-year PFS and OS rates were 61.4% ± 6.6% (95% CI, 48.4%–74.3%) and 68.6% (95% CI, 56.2%–80.6%), respectively. The validation cohort included 171 pediatric patients with the mean age of 8.1 ± 3.8 years (range: 1.4–17 years), eight of whom (4.7%) had metastatic disease (M+) at the time of diagnosis. The median follow-up period was 83.6 months (range: 5–227 months). The estimated 5-year PFS and OS rates were 65.2% (95% CI, 57.8%–72.6%) and 69.0% (95% CI, 61.7%–76.2%), respectively. A total of 59 patients were excluded. Eight normal cerebellums were obtained from 8 individuals without neurological disorders and with the mean age of 36.6 years (age range 0.5–58 years; male/female ratio 3:1).

**Table 1:** Clinicopathologic characteristics of two independent cohorts involved in this study.

Characteristics	Discovery cohort n = 81, (%)	Validation cohort n = 171, (%)
**Age at diagnosis**		
0–3 years	12 (14.8)	16 (9.4)
4–17 years	52 (64.2)	155 (90.6)
≥18 years	17 (21.0)	0 (0.0)
**Sex**		
Male	59 (72.8)	123 (71.9)
Female	22 (27.2)	48 (28.1)
**Tumor location**		
Vermis	22 (27.2)	59 (34.5)
Hemisphere	18 (22.2)	20 (11.7)
4^th^ ventricle	41 (50.6)	92 (53.8)
**Tumor size (cm)**		
≤ 4	24 (29.6)	51 (29.8)
> 4	57 (70.4)	120 (70.2)
**Metastasis**		
M0	81 (100.0)	163 (95.3)
M+	0 (0.0)	8 (4.7)
**Surgical resection**		
GTR	59 (72.8)	122 (71.3)
STR	22 (27.2)	49 (28.7)
**Histology**		
CMB	61 (75.3)	140 (81.9)
DNMB	12 (14.8)	17 (9.9)
LC/AMB	8 (9.9)	14 (8.2)
**Molecular subgroup**		
WNT	9 (11.1)	20 (11.7)
SHH	25 (30.9)	40 (23.3)
Group 3	13 (16.0)	49 (28.7)
Group 4	34 (42.0)	62 (36.3)
**CSI**		
Yes	67 (82.7)	159 (93.0)
No	14 (17.3)	12 (7.0)
**Chemotherapy**		
Yes	49 (60.5)	129 (75.4)
No	32 (39.5)	42 (24.6)
**Survival**		
Living	49 (60.5)	113 (66.1)
Dead	32 (39.5)	58 (33.9)

5hmC, 5-Hydroxymethylcytosine; CSI, craniospinal Irradiation; DN/EN, desmoplastic/nodular or extensive nodular; EOR, extent of resection; LC/A, large cell/anaplastic, MB, medulloblastoma; STR, subtotal removal.

### Decreased 5hmC Levels as an Epigenetic Hallmark of MB

**Figure 1:**
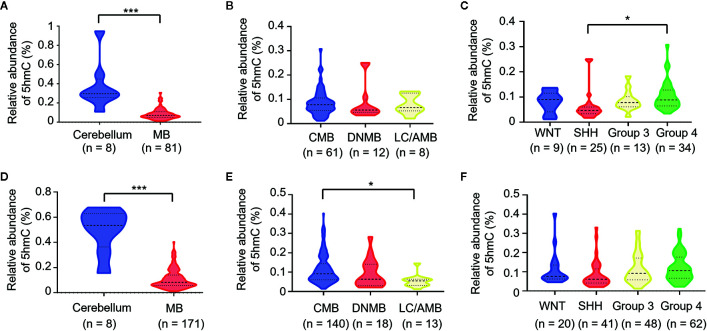
Loss of 5hmC as a hallmark in medulloblastomas. Comparative evaluation of global 5hmC levels as measured by UHPLC-MS/MS is analyzed between tumors and cerebellums, pathological subtypes, and molecular subgroups in the discovery cohort **(A–C)** and the validation cohort **(D–F)**, respectively. *P < 0.05, ****P < 0.0001, by unpaired t-test. CMB, classic medulloblastoma; DNMB, desmoplastic nodular medulloblastoma; LC/AMB, Large cell/anaplastic medulloblastoma.

We detected the relative abundances of 5hmC in MBs and normal cerebellums by UHPLC-MS/MS. In discovery cohort, global 5hmC levels were significantly lower in MBs than in the normal cerebellums (*P* < 0.0001, **Figure 1A**). Among four molecular subgroups, SHH-MBs had lower global 5hmC levels than Group 4-MBs (*P* = 0.038, **Figure 1C**). In the validation cohort, MBs had lower global 5hmC levels than normal cerebellums (*P* < 0.0001, **Figure 1D**). LC/AMBs had lower global 5hmC levels than CMBs (*P* = 0.027, **Figure 1E**). Moreover, somatic *TP53* mutations were detected in 38 pediatric SHH-MBs of validation cohort, three of which harbored a mutation in *TP53* exons (NM_000546, c.375G>A, c.454C>T, c.625_626delAG). No statistical difference in global 5hmC levels was observed between tumors with or without *TP5*3 mutation (0.050 *vs.* 0.083; *P* = 0.405).

### MBs With Low 5hmC Levels Had Poor Survival Rates

**Figure 2:**
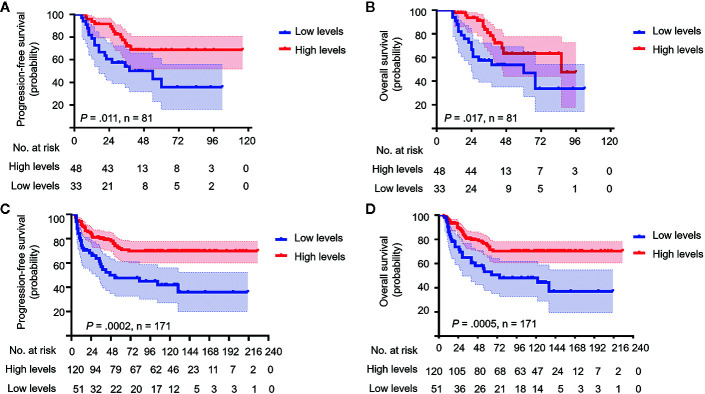
Medulloblastomas with low**** global ****5hmC levels show poor prognosis. Kaplan-Meier plots of estimated overall survival (OS) and progression-free survival (PFS) time distributions stratified by global 5hmC levels (low 5hmC levels *vs.* high 5hmC levels) are respectively analyzed in the discovery cohort (n = 81) **(A, B)** and the validation cohort (n = 171) **(C, D)**. Survival differences are calculated using continuous log-rank test. The numbers below the X-axis indicate the number of persons at risk at each time point.

To determine whether 5hmC could be a prognostic predictor in MB, we compared the PFS and OS stratified by global 5hmC levels. In the discovery cohort, MBs with low global 5hmC levels were associated with lower 5-year PFS and OS rates than those with high global 5hmC levels (5-year PFS: 35.8% ± 10.7% *vs.* 66.5 ± 7.5%, *P* = 0.011; 5-year OS: 47.0% ± 10.0% *vs.* 62.4% ± 8.9%, *P* = 0.017; **Figures 2A, B**). In the validation cohort, MBs with low 5hmC levels also showed worse OS and PFS compared to those with high 5hmC levels (5-year PFS: 44.8% ± 7.7% *vs.* 72.1% ± 4.2%, *P* = 0.0002; 5-year OS: 56.4% ± 7.8% *vs.*74.6% ± 4.2%, *P* = 0.0005; **Figures 2C, D**).

We performed Harrell’s C index and time-dependent ROC curves to assess the discriminatory capacity of 5hmC-based classification and molecular classification as prognostic biomarkers for survival in both cohorts. C-index showed that 5hmC-based classification had good predictive accuracy in terms of 5-year PFS and OS (range: 0.598–0.630), as did the molecular subgroup (range: 0.568–0.603; **Supplementary Table S1**). Time-dependent ROC curves also confirmed the similar AUCs for 5-year and 8 (10)-year survival between 5hmC-based classification (range: 0.596–0.641) and the molecular classification in both cohorts (range: 0.575–0.624; **Supplementary Figure S1**). These results indicate that 5hmC could be a potential biomarker for prognostic prediction in MB.

### 5hmC Was an Independent Prognostic Indicator for MB

To further identify the prognostic value of 5hmC in MBs, univariate and multivariate Cox regression analyses were performed in both cohorts. In the discovery cohort, multivariate Cox regression analysis identified global 5hmC levels (low *vs.* high) as a significant predictor of PFS (HR = 3.711, 95% CI = 1.648–7.357, *P* = 0.003) and OS (HR = 3.974, 95% CI = 1.641–8.394, *P* = 0.002) (**Table 2**). Meanwhile, molecular subgroup (WNT, SHH, Group 3, and Group 4), histological subtype (CMB, DNMB, and LC/AMB), and CSI treatment (yes *vs.* no) were identified as prognostic predictors (**Table 2**). In validation cohort, multivariate analysis confirmed that global 5hmC levels (PFS: HR = 2.830, 95% CI = 1.623–4.660, *P* = 0.0002; OS: HR = 2.529, 95% CI = 1.475–4.337, *P* = 0.001), as well as metastatic status, CSI treatment, histological subtype, and molecular subgroup, contributed significantly PFS and OS (**Table 3**).

**Table 2:** Cox proportional hazards models for progression-free survival and overall survival rate in discovery cohort of medulloblastomas (n = 81).

Variables	Progression-free survival			Overall survival		
	HR	95% CI	*P*	HR	95% CI	*P*
**Univariate analysis**						
5hmC levels, low *vs.* high	2.498	1.092–4.422	.017	2.521	1.134–4.751	.010
Pathological subtype						
CMB *vs.* LC/A	0.210	0.088–0.501	.001	0.323	0.138–0.804	.012
DNMB *vs.* LC/A	0.134	0.077–0.289	.002	0.152	0.096–0.435	.006
CSI, yes *vs.* no	0.321	0.140–0.727	.005	0.349	0.163–0.749	.007
Molecular subgroup						
WNT *vs.* Group 3	0.092	0.011–0.752	.024	0.103	0.039–0.666	.011
SHH *vs.* Group 3	0.310	0.117–0.820	.018	0.274	0.101–0.741	.019
Group 4 *vs.* Group 3	0.490	0.211–1.138	.097	0.473	0.175–0.948	.041
Chemotherapy, yes *vs.* no	0.892	0.440–1.808	.751	0.579	0.254–1.320	.193
EOR, GTR *vs.* STR	1.004	0.469–2.193	.971	0.941	0.432–2.051	.879
Location, CH *vs.* midline	0.932	0.370–2.352	.882	1.203	0.485–2.981	.690
Tumor size (cm), ≥ 4 *vs.* <4	1.389	0.621–3.110	.424	1.372	0.609–3.089	.446
Gender, male *vs.* female	1.536	0.573–2.120	.960	0.914	0.420–1.988	.821
Age (years)						
3–17 *vs.* 0–3	0.958	0.326–2.577	.746	0.899	0.301–2.703	.759
≥18 *vs.* 0–3	0.582	0.174–1.941	.085	0.477	0.141–1.613	.234
**Multivariate analysis**						
5hmC levels, low *vs.* high	3.711	1.648–7.357	.003	3.974	1.641–8.394	.002
Molecular subgroup						
WNT *vs.* Group 3	0.086	0.037–0.715	.023	0.075	0.039–0.622	.016
SHH *vs.* Group 3	0.472	0.107–1.068	.065	0.284	0.090–0.895	.032
Group 4 *vs.* Group 3	0.512	0.216–1.215	.129	0.563	0.178–0.980	.045
Pathological subtype						
CMB *vs.* LC/AMB	0.215	0.103–0.715	.008	0.393	0.144–1.073	.066
DNMB *vs.* LC/AMB	0.135	0.098–0.454	.006	0.093	0.111–0.795	.015
CSI, yes *vs.* no	0.636	0.270–0.566	.016	0.507	0.150–1.412	.054
Age (year)						
3–17 *vs.* 0–3	1.286	0.367–4.512	.694	0.962	0.285–3.243	.950
≥18 *vs.* 0–3	0.280	0.125–2.414	.429	0.687	0.159–2.968	.615
Resection, GTR *vs.* STR	1.018	0.365–2.841	.973	0.987	0.454–2.135	.726
Chemotherapy, yes *vs.* no	1.940	0.637–4.910	.307	1.743	0.644–3.776	.563

5hmC, 5-Hydroxymethylcytosine; CH, cerebellar hemisphere; CSI, craniospinal Irradiation; DN/EN, desmoplastic/nodular or extensive nodular; EOR, extent of resection; LC/A, large cell/anaplastic, MB, medulloblastoma; STR, subtotal removal.

**Table 3:** Cox proportional hazards models for progression-free survival and overall survival rate in validation cohort of medulloblastomas (n = 171).

Variables	Progression-free survival			Overall survival		
	HR	95% CI	*P*	HR	95% CI	*P*
**Univariate analysis**						
5hmC levels, low *vs.* high	2.711	1.632–4.501	.001	2.555	1.517–4.302	.0004
Metastatic status, M+ *vs.* M0	5.628	2.199–14.402	<.0001	8.018	3.091–20.798	<.0001
CSI, yes *vs.* no	0.269	0.136–0.480	.0002	0.262	0.141–0.588	.0001
Molecular subgroup						
WNT *vs.* Group 3	0.223	0.067–0.721	.013	0.145	0.034–0.611	.009
SHH *vs.* Group 3	0.403	0.173–0.920	.028	0.426	0.210–0.864	.014
Group 4 *vs.* Group 3	0.543	0.303–0.974	.041	0.534	0.295–0.966	.038
Pathological subtype						
CMB *vs.* LC/AMB	0.220	0.117–0.430	.0002	0.255	0.104–0.422	.0003
DNMB *vs.* LC/AMB	0.188	0.050–0.513	.002	0.216	0.052–0.439	.005
Age (year), 3–17 *vs.* 0–3	0.383	0.194–0.757	.006	0.476	0.266–1.007	.052
EOR, GTR *vs.* STR	0.762	0.446–1.300	.319	0.807	0.462–1.412	.453
Chemotherapy, yes *vs.* no	0.929	0.518–1.665	.804	0.993	0.551–1.789	.981
Location, CH *vs.* midline	1.083	0.821–1.428	.574	1.066	0.802–1.418	.658
Tumor size (cm), <4 *vs.* ≥ 4	1.383	0.781–2.450	.266	0.806	0.452–1.438	.465
Gender, male *vs.* female	0.926	0.534–1.606	.784	0.948	.537–1.673	.854
**Multivariate analysis**						
5hmC levels, Low *vs.* high	2.830	1.623–4.660	.0002	2.529	1.475–4.337	.001
Metastatic status, M+ *vs.* M0	10.056	3.896–21.379	<.0001	11.300	4.097–31.800	.032
CSI, yes *vs.* no	0.389	0.147–0.561	.028	0.420	0.197–0.896	.011
Pathological subtype						
CMB *vs.* LC/AMB	0.447	0.214–0.935	.018	0.378	0.185–0.772	.008
DN/ENMB *vs.* LC/AMB	0.479	0.121–1.466	.131	0.454	0.120–1.176	.096
Molecular subgroup						
WNT *vs.* Group 3	0.277	0.082–0.869	.027	0.139	0.031–0.628	.009
SHH *vs.* Group 3	0.342	0.157–0.659	.002	0.319	0.128–0.792	.011
Group 4 *vs.* Group 3	0.610	0.362–1.058	.075	0.564	0.307–1.038	.087
EOR, GTR *vs.* STR	1.094	0.635–2.012	.572	1.014	0.602–2.054	.689
Chemotherapy, yes *vs.* no	1.126	0.593–2.027	.337	1.072	0.566–2.028	.763
Age (years), 3–17 *vs.* 0–3	0.967	0.343–2.732	.949	1.138	0.352–3.307	.895

5hmC, 5-Hydroxymethylcytosine; CH, cerebellar hemisphere; CSI, craniospinal Irradiation; DN/EN, desmoplastic/nodular or extensive nodular; EOR, extent of resection; LC/A, large cell/anaplastic, MB, medulloblastoma; STR, subtotal removal.

### Nomograms Showed the Relative Utility of Variables in Predicting OS and PFS

**Figure 3:**
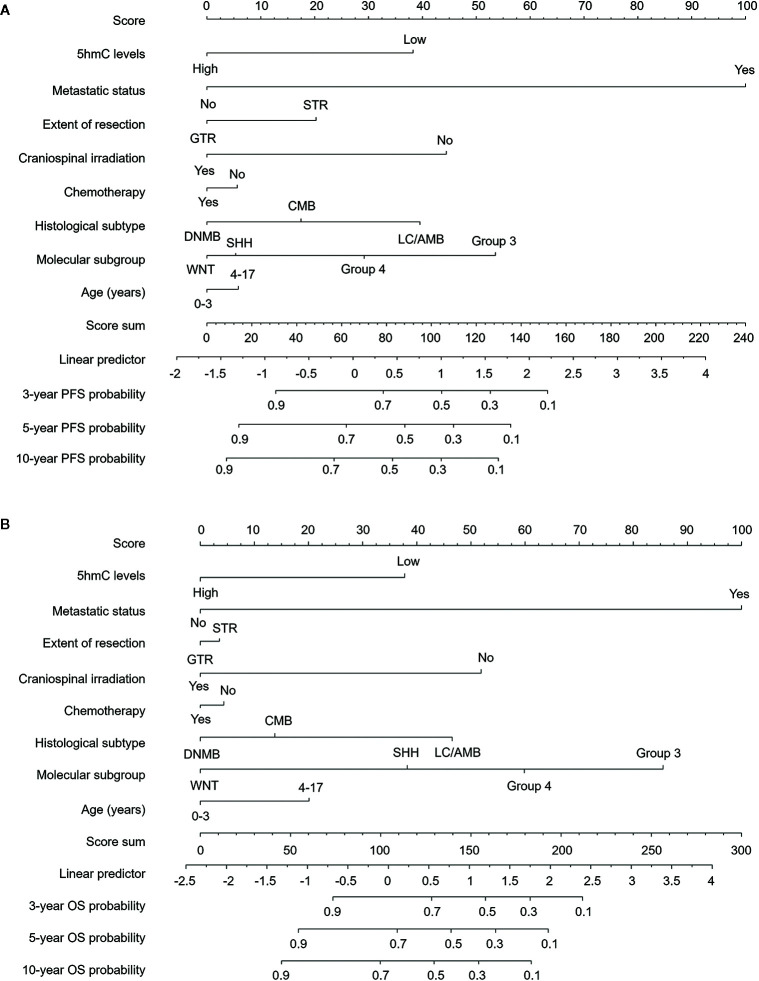
****Survival nomograms for pediatric medulloblastoma. Nomograms are created based on the multivariable Cox model of the validation cohort. The presence or absence of each variable is scored (top row). The cumulative score from each variable is used to calculate 3-year, 5-year, or 10-year PFS **(A)** and OS **(B)** probabilities. GTR, gross total resection; STR, subtotal resection; DNMB, Desmoplastic/nodular medulloblastoma; LC/AMB, Large cell/anaplastic medulloblastoma; OS, overall survival; PFS, progression-free survival.

**Figure 4:**
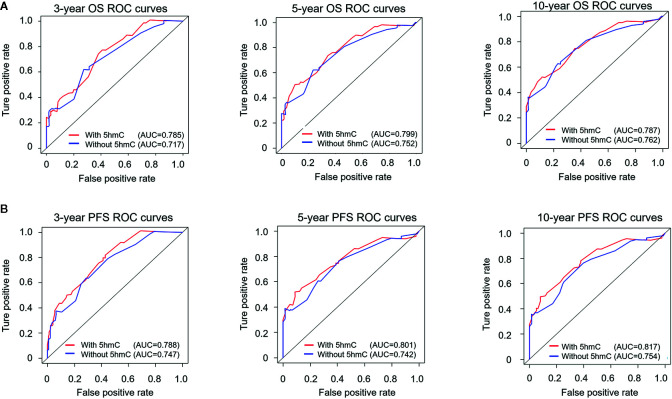
Time-dependent receiver operating characteristic (ROC) analysis of OS **(A)** and PFS **(B)** for nomograms with or without 5hmC-based classification. The prognostic accuracy is analyzed by area under the ROC curves (AUCs) at 3, 5, and 10 years. OS, overall survival; PFS, progression-free survival.

We generated survival nomograms to show the relative clinical effect of each variable to predict 3-year, 5-year, and 10-year PFS and OS based on multivariate Cox model of the validation cohort (**Figures 3A, B**). The calibration plots showed the acceptable agreement between nomogram-based predictions and clinical observations (**Supplementary Figure S2**). We then respectively compared the predictive accuracies of survival between nomograms with 5hmC and without 5hmC using time-dependent ROC curves (**Figure 4**). We found that AUCs of 3-, 5, 10-year OS and PFS (range: 0.785–0.817) were higher in nomograms with 5hmC-based classification than those without 5hmC-based classification (range: 0.717–0.762). These results indicate that integration of 5hmC-based classification with clinical and molecular factors could improve risk stratification for patients with MB.

### Loss of 5hmC Was Linked to High Cell Proliferation in MBs

**Figure 5:**
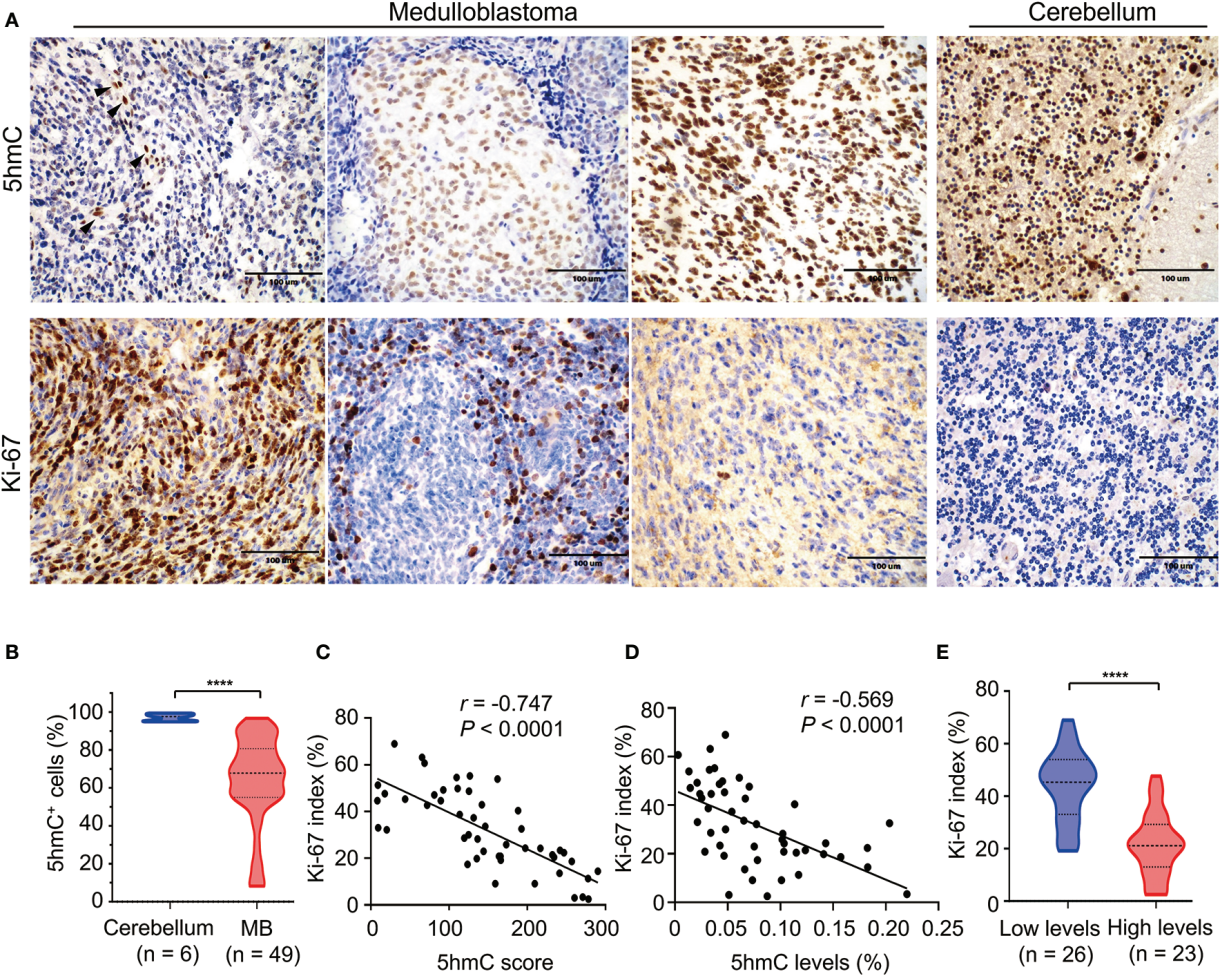
The relationship between 5hmC and cell proliferation in medulloblastomas. **(A)** Representative images of 5hmC immunoreactivity and Ki-67 immunoreactivity in medulloblastomas (400×). Non-tumor cells (endothelial cells) in the microenvironment were used as a positive-control tissue (black arrow). Scale bars represent 100 μM. **(B)** Comparative evaluation of nuclear positivity of 5hmC antibody between tumors and normal cerebellums. **(C)** A significant inverse correlation between 5hmC score and Ki-67 index (n = 49). Data are analyzed using Pearson correlation coefficient. **(D)** A significant inverse correlation between the relative abundance of 5hmC and Ki-67 index (n = 49). Data are analyzed using Pearson correlation coefficient. **(E)** Comparative evaluation of Ki-67 index between medulloblastomas with low and high 5hmC levels. ****P < 0.0001, by unpaired t-test.

We performed the IHC for 5hmC staining on 49 pediatric MB samples in the validation cohort containing four molecular subgroups to further confirm the reduction of 5hmC generation in MB. We observed that MBs presented lower nuclear positivity and staining intensity of 5hmC antibody compared with normal cerebellums (*P* < 0.0001; **Figures 5A, B**). We then performed IHC for Ki-67 staining to determine the relationship between 5hmC and cell proliferation. We found that Ki-67 index reversely correlated with 5hmC score (*r* = -0.747, *P* < 0.0001, **Figures 5A, C**) and 5hmC levels (*r* = -0.569, *P* < 0.0001, **Figure 5D**), respectively. Moreover, MBs with low 5hmC levels had higher Ki-67 index than those with high 5hmC levels (*P* < 0.0001; **Figure 5E**).

### Loss of 5hmC Related to Low Nuclear TET1/2 Expression

**Figure 6:**
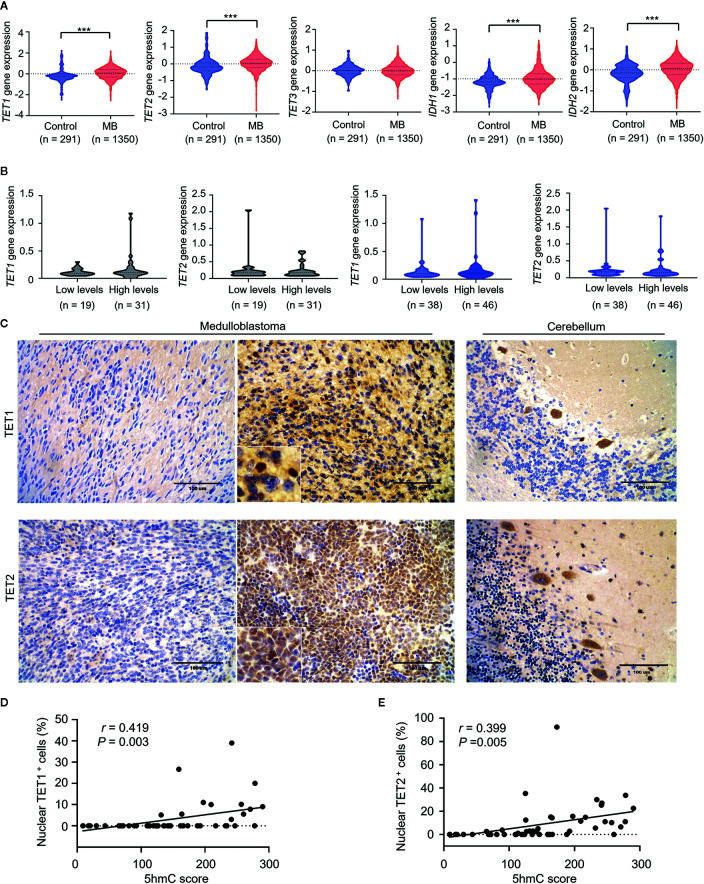
Loss of 5hmC in medulloblastomas is associated with decreased nuclear expression of TET1/2 proteins. **(A)** The comparative evaluation of mRNA expression levels of *TET1/2/3* and *IDH1/2* between MB samples (n = 1350) and normal cerebellums (n = 291). ***P < 0.001, by unpaired t-test. **(B)** The comparative evaluation of mRNA expression levels of *TET1/2* between MBs with low or high global 5hmC levels in the discovery cohort (n = 50) and the validation cohort (n = 84). Data are analyzed using unpaired t-test. **(C)** Representative imaging of IHC for TET1/2 staining in MBs (left, nuclear negative; right, nuclear positive) and in normal cerebellums. **(D)** A strong correlation between TET1 nuclear staining and 5hmC score (n = 49). Data are analyzed using Pearson correlation coefficient. **(E)** A strong correlation between TET2 nuclear staining and 5hmC score (n = 49). Data are analyzed using Pearson correlation coefficient.

Since changes in expression of *TET* and *IDH* genes were linked to altered 5hmC levels in cancer (19), we determined whether the loss of 5hmC in MB was caused by abnormal expression of TET or IDH genes. Firstly, we found that gene mutations in *TET1/2/3* or *IDH1/2* were extremely rare in MBs (range: 0%–4.3%; **Supplementary Table S2**). In mRNA-expression levels, we found that the levels of *TET1/2* and *IDH1/2* were higher in MBs (n = 1350) than in normal cerebellums (n = 291; all *P* < 0.001; **Figure 6A**). We then detected the mRNA-expression levels of *TET1*/*2* on our MB samples in both cohorts using the QGP analysis. We found no significant differences of *TET1*/*2* expression between high-5hmC and low-5hmC MBs (**Figure 6B**). To determine the relationship between 5hmC and expression of TET1/2 proteins, we performed IHC staining for TET1/2 antibodies in MB samples (**Figure 6C**). Interestingly, we observed a significant association between 5hmC scores and nuclear positivity for TET1/2 in MB samples (*r* = 0.419, *P* = 0.003; *r* = 0.399, *P* = 0.005; **Figures 6D, E**).

## Discussion

Recent advances have revealed molecular and clinical heterogeneities in MB (8, 37). Therefore, identifying reliable biomarkers is crucial for tailoring individual treatment strategies of patients with MB. In this study, we investigated the clinical relevance of a novel epigenetic biomarker—5hmC, in two large independent MB cohorts. We demonstrated that loss of 5hmC is a common epigenetic event in MBs. More importantly, we provided the first evidence that loss of 5hmC significantly correlate with poor survival in patients with MB. Our findings suggest that the integration of 5hmC-based classification in existing risk stratification models can facilitate the individualized therapy of MB in future clinical trials.

Previous studies reported that reduced global 5hmC levels correlated with a more aggressive phenotype and poorer survival in glioma (25, 26, 38). In this study, we determined global 5hmC levels as a significant prognostic indicator for patients with MB, independently of clinical or molecular parameters. This implies that characterization of 5hmC levels could segregate individuals with MB into groups with favorable or extremely poor survival, where those with low 5hmC levels may need more clinical care. Moreover, given the important prognostic value of 5hmC, it is interesting to consider the functional relevance of this epigenetic biomarker in MB tumorigenesis. The loss of 5hmC in MB indicates an imbalance occurred between methylation and demethylation, which may lead to tumor-suppressor gene silencing or oncogene activation (39, 40). More importantly, a recent study of ovarian cancer demonstrated that the pharmacologic reversal in 5hmC levels using DNA methyltransferase inhibitors (DNMTIs)—5-azacytidine, could enhance the chemosensitivity of platinum resistant tumors and prolong survival *in vitro* and *in vivo* (41). This finding provides a novel hint that MB patients with 5hmC loss may benefit from treatment of DNMTIs. Thus, future genome-wide hydroxymethylation studies are urgently needed to improve our understanding of potential tumorigenic mechanisms caused by altered 5hmC levels and to investigate the potential new therapies for MB.

Interestingly, we observed that MBs with low 5hmC levels had higher cell proliferation. This finding is consistent with a previous study showing the inverse link between 5hmC levels and cell proliferation in multiple human cancers (24). The higher Ki-67 index indicates more aggressive biological behavior and shorter survival in brain tumors (42–44). More importantly, we previously showed Ki-67 index as an independent prognostic predictor in MBs (29). These data may offer a plausible explanation for the poor survival of MBs with low global 5hmC levels. Moreover, we observed that strong 5hmC staining could be seen not only in the well-differentiated, non-proliferating cell types of the normal cerebellum, but also in neuronal differentiated cells within nodular areas of DNMB. In contrast, poorly differentiated cells intervening nodular areas exhibited loss of 5hmC and higher proliferation. This finding indicates that loss of 5hmC may lead to the abnormal differentiation of MB cells, considering that TET activity and 5hmC levels are necessary for successful tissue differentiation (45).

Survival nomograms has been proposed as a new alternative of traditional risk classification system in multiple types of cancers due to its predictive accuracy and convenient utility (46, 47). In this study, we developed a survival nomogram based on global 5hmC levels, molecular subgroup and clinico- pathological factors to predict outcome in pediatric patients with MB. To the best of our knowledge, this is the first survival nomogram for pediatric MB that integrates comprehensive prognostic predictors based on a large patient cohort. Our nomogram performed well in predicting survival, and its prediction was supported by the C-index and the calibration curve. We believe that this model can provide an individualized survival prediction for both physicians and patients through this easy-to-use scoring system.

The regulation of DNA hydroxymethylation is mediated by several factors. TET1–3 proteins are responsible for the conversion of 5mC into 5hmC through the oxidation reaction, and therefore, their expressions tend to be strongly responsible for the formation of 5hmC marks (18, 19). Moreover, mutated Isocitrate dehydrogenases (IDHs) can convert the TET co-substrate α-Ketoglutarate into R-2-hydroxyglutarate (R-2HG), which can act as a competitive inhibitor of the TET protein family. Previous studies demonstrated that dysfunctional *TET* and/or *IDH* genes influence the regulation of DNA hydroxymethylation and lead to reduction of 5hmC generation in cancers (19). However, the gene mutations of *TET1/2/3* and *IDH1/2* were extremely rare in MBs (48–51), while the RNA levels of *TET1/2* and *IDH1/2* were higher in MBs than in normal cerebellums. These data indicate that genetic alterations may be not the cause of 5hmC loss in MB. Interestingly, we found that reduced 5hmC in MBs relate to decreased nuclear expression of TET1/2 proteins, suggesting that the subcellular localization or nuclear inactivation of TET1/2 proteins may be responsible for the altered 5hmC levels in MBs. Future studies are needs to elucidate the potential mechanisms that inhibit the activity of TET1/2 proteins in MBs.

There were some limitations of this study. As a retrospective review, it was subjected to selection and recall bias. A second limitation is that several important prognostic indicators in MB, such as the MYC or MYCN amplifications, were not included in our risk stratification models. To overcome this limitation, future studies are required to determine the relationships between global 5hmC levels and the statuses of these biomarkers, and whether the accuracy of survival prediction could be improved by incorporating global 5hmC levels with these biomarkers for risk stratification in patients with MB. In addition, detecting 5hmC with IHC staining could be more practical in clinical applications than with UHPLC-MS/MS assays. Additional large-scale, multi-centered retrospective studies are needed to fully evaluate the utility of 5hmC staining as a prognostic tool.

Taken together, our data demonstrate that loss of 5hmC is associated with aggressive behavior and poor prognosis in MB. Our study highlights 5hmC as a novel prognostic indicator that can be used in clinic to improve the accuracy of survival prediction for patients with MB. Additional large-scale, multi-centered retrospective and prospective studies are still required to evaluate the utility of 5hmC as a prognostic tool in future clinical trials.

## Data Availability Statement

The raw data supporting the conclusions of this article will be made available by the authors, without undue reservation.

## Ethics Statement

The studies involving human participants were reviewed and approved by the Ethics Review Board of Beijing Tian Tan Hospital. Written informed consent to participate in this study was provided by the participants’ legal guardian/next of kin.

## Author Contributions

The study was conceived and designed by FZ, PL, and YN. Clinical data collection was performed by CL, SZ, and HZ. Sample collection was performed by CL. Bioinformatics and statistical analyses were performed by JZ and Z-WZ. Histopathological diagnosis was performed by W-MT and LL. FZ, Z-WZ, YC, and JZ conducted the experiments of UHPLC-MS/MS and IHC. The manuscript was written and reviewed by FZ and YN. All authors contributed to the article and approved the submitted version.

## Funding

This study was supported by the National Natural Science Foundation of China under Grant number 81902862 (FZ); the Capital Health Development and Research Special Projects of Beijing under Grant number 2018-2-1073 (CL); the Non-profit Central Research Institute Fund of Chinese Academy of Medical Sciences under Grant numbers 2016ZX310182-2 and 2016ZX310176-6 (YN); the Medical Epigenetics Research Center and Chinese Academy of Medical Sciences under Grant number 2019PT310017, to YN]; the Beijing Dongcheng District Outstanding Talent Funding Project under Grant number 2019DCT-M-16 (FZ).

## Conflict of Interest

The authors declare that the research was conducted in the absence of any commercial or financial relationships that could be construed as a potential conflict of interest.
