# Supplementary material for: Loss of 5-Hydroxymethylcytosine as an Epigenetic Signature That Correlates With Poor Outcomes in Patients With Medulloblastoma
Source: Front Oncol. 2021 Feb 24;11:603686. doi: 10.3389/fonc.2021.603686 (PMC7945595; doi:10.3389/fonc.2021.603686)
Supplement: Supplementary file 1 [file DataSheet_1.pdf]

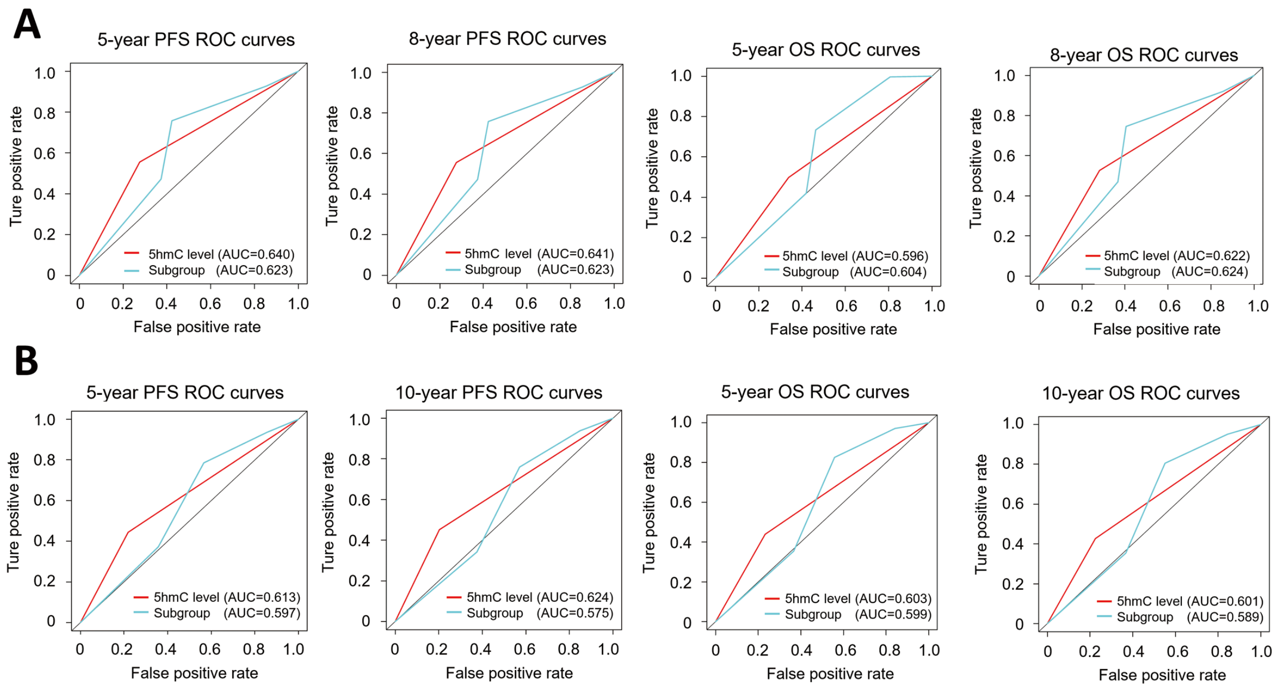

Supplementary Figure S1 (Related to Figure 2). Time-dependent receiver operating characteristic (ROC) analysis of 5 and 8 (10)-year overall survival (OS) and progression-free survival (PFS) prediction for 5hmC levels and molecular subgroup in discovery cohort **(A)** and validation cohort **(B)**, respectively. The prognostic accuracy is analyzed by area under the ROC curves (AUCs) at 5 and 8 (10) years. OS = overall survival, PFS = progression-free survival.

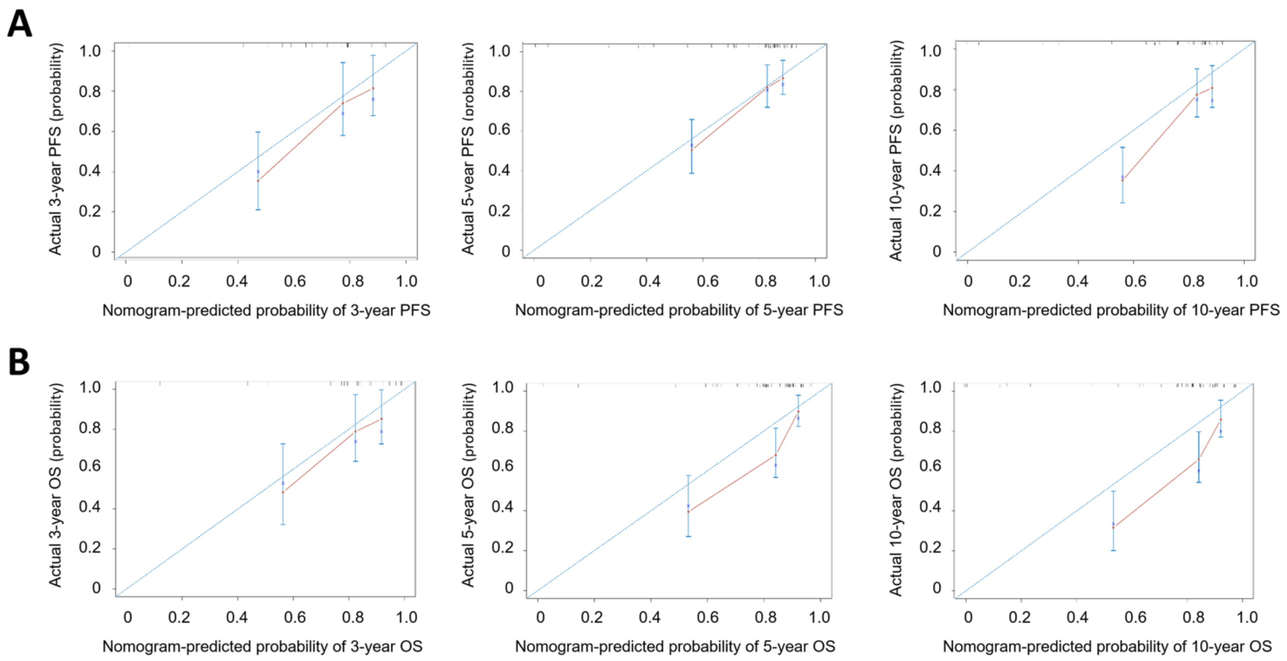

Supplementary Figure S2 (Related to Figure 3). The calibration curves for predicting PFS **(A)** and OS **(B)** at 3, 5 and 10 years. The actual survival is plotted on the y-axis, and nomogram-predicted probability is plotted on the x-axis. The model performance is shown relative to the 45° line, representing the performance of an ideal nomogram for which the predicted outcome corresponding with actual outcome. OS = overall survival, PFS = progression-free survival.

Supplementary Table S1. C-index analysis for 5-year progression-free survival and overall survival in discovery and validation cohorts

	Progression-free Survival		Overall Survival	
	C-Index	95% CI	C-Index	95% CI
Discovery cohort				
5hmC-based classification	0.619	0.531-0.707	0.630	0.542-0.718
Molecular subgroup	0.603	0.503-0.691	0.575	0.497-0.673
Validation cohort				
5hmC-based classification	0.604	0.540-0.667	0.598	0.535-0.661
Molecular subgroup	0.577	0.512-0.642	0.568	0.501-0.635

Supplementary Table S2. The mutation information of 5hmC-related genes in medulloblastoma

Gene	Studies	Somatic Mutation	Sample ID	Mutation type	Protein change	Location	Annotation
TET1	Nature 2016[1]	2.2%	MB- REC-26	Missense	S1682R	TET-JBP	Unknown
	Nature 2012[2]	0.8%	ICGC- MB49	Truncating	R276	/	Unknown
TET2	Nature 2016[1]	2.2%	MB- REC-26	Truncating	R1452	TET-JBP	Unknown
				Missense	R1966C	/	Unknown
TET3	Nature 2016[1]	4.3%	MB- REC-26	Truncating	R899	TET-JBP	Unknown
			MB- REC-15	Missense	R1609L	/	Unknown
IDH1	Nature 2012[2]	1.1%	MD-278	Missense	R132C	Iso-dh	Oncogenic mutation

Abbreviations: Iso-dh, Isocitrate /isopropylmalate dehydrogenase; TET-JBP: Oxygenase domain of the 2OGFeDO superfamily.

No *IDH2* mutation was observed in sequencing data. Data source: http://www.cbioportal.org.

References:

1. Lin CY, Erkek S, Tong Y, et al. Active medulloblastoma enhancers reveal subgroup-specific cellular origins. Nature. 2016; 530(7588):57-62.

2. Jones DT, Jager N, Kool M, et al. Dissecting the genomic complexity underlying medulloblastoma. Nature. 2012; 488(7409):100-105.
